# Combinatorial transient gene expression strategies to enhance terpenoid production in plants

**DOI:** 10.3389/fpls.2022.1034893

**Published:** 2022-12-13

**Authors:** Soyoung Park, Vimalraj Mani, Jin A. Kim, Soo In Lee, Kijong Lee

**Affiliations:** Department of Agricultural Biotechnology, National Institute of Agricultural Sciences, Rural Development Administration, Jeonju, Republic of Korea

**Keywords:** MEP, MVA, terpenoid, agroinfiltration, transient expression, linalool, costunolide

## Abstract

**Introduction:**

The monoterpenoid linalool and sesquiterpenoid costunolide are ubiquitous plant components that have been economically exploited for their respective essential oils and pharmaceutical benefits. In general, monoterpenes and sesquiterpenes are produced by the plastid 2-C-methyl-D-erythritol 4-phosphate (MEP) and cytosolic mevalonate (MVA) pathways, respectively. Herein, we investigated the individual and combinatorial potential of MEP and MVA pathway genes in increasing linalool and costunolide production in *Nicotiana benthamiana*.

**Methods:**

First, six genes from the MEP (1-deoxy-D-xylulose-5-phosphate synthase, 1-deoxy-D-xylulose 5-phosphate reductoisomerase, 4-diphosphocytidyl-2-C-methyl-D-erythritol kinase, geranyl pyrophosphate synthase, and linalool synthase) and MVA (acetoacetyl-CoA-thiolase, hydroxy-3-methylglutaryl-CoA reductase, farnesyl pyrophosphate synthase, germacrene A synthase, germacrene A oxidase, and costunolide synthase) pathways were separately cloned into the modular cloning (MoClo) golden gateway cassette. Second, the cassettes were transformed individually or in combination into the leaves of *N. benthamiana* by agroinfiltration.

**Results and discussion:**

Five days post infiltration (DPI), all selected genes were transiently 5- to 94-fold overexpressed. Quantification using gas chromatography-Q-orbitrap-mass spectrometry (GC-Q-Orbitrap-MS) determined that the individual and combinatorial expression of MEP genes increased linalool production up to 50–90ng.mg^-1^ fresh leaf weight. Likewise, MVA genes increased costunolide production up to 70–90ng.mg^-1^ fresh leaf weight. Our findings highlight that the transient expression of MEP and MVA pathway genes (individually or in combination) enhances linalool and costunolide production in plants.

## 1 Introduction

Terpenoids are a source of natural products that has a variety of essential and non-essential functions in metabolism, and abiotic and biotic factor interactions. They are used in the food and pharmaceutical industries as medications, chemical feedstocks, and food additives (Mani et al., 2021). Contemporary elicitors, such as abiotic and biotic elicitors, are also employed to activate secondary metabolism pathways and enhance the production of target terpenoids (Boutanaev et al., 2015). The mevalonate (MVA) and plastidial 2-C-methyl-D-erythritol 4-phosphate (MEP) pathways are shared by a majority of eukaryotes, such as fungi, animals, and higher plants, are involved in the production of terpenoids. Despite their structural diversity, the fundamental components of all terpenoids are isopentyl pyrophosphate (IPP) and dimethylallyl pyrophosphate (DMAPP) (Dudareva et al., 2005; Lombard and Moreira, 2011; Banerjee and Sharkey, 2014). The widespread use of terpenoids has generated significant economic value to date; however, enhancing the efficiency of exploitation, particularly for wild resources, remains a significant problem.

Several economically significant terpenoids are found in trace amounts in nature, and metabolic engineering, or, in a more modern sense, synthetic metabolic pathways has emerged as an intriguing method for expanding terpenoid synthesis (Zhang and Hong, 2020). Some of the frequently employed techniques include overexpression of rate-limiting enzymes, often including 1-deoxy-D-xylulose 5-phosphate synthase (DXS), 1-deoxy-D-xylulose-5-phosphate reductoisomerase (DXR), and 3-hydroxy-3-methylglutaryl-CoA reductase (HMGR), reconstruction of the synthetic MVA pathway in *Escherichia coli* and overexpression of a transcription factor to increase the overall flux (Paddon et al., 2013; Lv et al., 2016). The rate-limiting enzyme of MEP (DXS and DXR) and MVA (HMGR) activity is highly regulated and considered in the upstream biosynthetic pathway for the synthesis of diverse functional metabolites (Wei et al., 2019). Typically, the overexpression of these important genes is enough to double the concentration of target compounds. Hence, we overexpressed six MEP and six MVA genes in *Nicotiana benthamiana* plants using a modular cloning (MoClo) system to enhance linalool and costunolide production.

More than 80% of essential oils are composed of monoterpenes, a terpene family comprised of two isoprene units with the chemical formula C_10_H_16_. All monoterpenes are synthesized by the geranyl pyrophosphate synthase (GPPS) enzyme from the precursor of geranyl pyrophosphate (GPP). Monoterpenes have either an acyclic (linear) or cyclic (comprising rings) structure (Lombard and Moreira, 2011; Pereira et al., 2018). Monoterpenes can also contain double bonds, acetoxy, carbonyl, hydroxyl, or other substituents (Lücker et al., 2002). Monoterpenes have widespread applications as flavorings and fragrances in food. Furthermore, owing to their anti-inflammatory, analgesic, and wound-healing effects they are widely applied in cosmetics industry and pharmaceutical products (Aprotosoaie et al., 2014; Herman et al., 2016). The MEP pathway generates linalool in plants through linalool synthase. Over 200 dicotyledonous and monocotyledonous plant species found worldwide contain essential oils, including linalool. Linalool is mostly found in the Apiaceae (genus *Coriandrum*), Lauraceae (genus *Cinnamomum*), and Lamiaceae (genus *Lavandula*) plant families. Linalool may predominate in the composition of certain plant species, depending on a variety of variables such as plant part, harvest season, regional temperature, extracting process, and abiotic factors that impact the plant species chemotype. (Pereira et al., 2018).

Sesquiterpene lactones (SLs) constitute a prominent family of secondary metabolites in plants and over 4000 distinct structures have been identified. Most of these odorless typically sweet – sour semi-polar chemicals are functional components of a number of therapeutic plants used in (ancient) medicine. (Zhang et al., 2005). The precursor of all SLs is farnesyl pyrophosphate (FPP), which is produced by the farnesyl pyrophosphate synthase (FPS) enzyme. A popular SL of the germacranolide class is costunolide. The chemical formula of this substance, which appears in the form of a white crystalline powder, is C_15_H_20_O_2_. The costus root (*Saussurea lappa* Clarke), lettuce (*Aucklandia lappa*), and several other plant species were the original sources of this chemical (Chadwick et al., 2013). As enzymes exist in chicory roots that convert FPP into costunolide, De Kraker et al. first postulated the presence of a route creating costunolide from FPP. (De Kraker et al., 2002). First and foremost, germacrene A synthase (GAS) converts FPP to germacrene A (De Kraker et al., 1998). *Artemisia annua*, chicory, lettuce, and feverfew are just a few of the *Asteraceae* plants from which GAS genes were identified and described (Bouwmeester et al., 2002; Bennett et al., 2002; Bertea et al., 2006). In the subsequent phase within the pathway, germacrene A oxidase (GAO) oxidizes germacrene A at its C_13_ methyl to produce germacra-(10), “4,11(13)-trien-12-ol,” which is further oxidized to produce germacra-1(10), 4,11(13)-trien-12-al, and germacra-1(10), 4,11(13)-trien-12-oic acid (De Kraker et al., 2002). Germacra-1(10), trien-12-oic acid is hydroxylated at the C6 position by suspected cytochrome P450 mono-oxygenase, and subsequently the C_12_ carboxylic and C_6_ hydroxyl groups spontaneously cyclize to form costunolide (Liu et al., 2011).

Major advances in synthetic biology has opened up a diverse important role in many applications in the past ten years for the synthesis of terpenoids in heterologous hosts. Heterologous biosynthesis of natural products in microorganisms or plants is one of the most active areas of research in the field of synthetic biology, which significantly reduces natural poverty, environmental pollution, and economic costs (Cravens et al., 2019; Zhu et al., 2021). These challenges can be addressed by engineering terpenoid biosynthesis on heterologous host production platforms. In particular, plant-based synthesis processes are preferred because they have the compartmentalization as well as enzymatic reactions, which facilitate the exchange of functional routes across other plants. Model tobacco plants (*N. tabacum* and *N. benthamiana*) have been widely used over the last decade to reconstitute biosynthetic pathways for natural plant products. The main reason for selecting particular plant species is that it is extremely susceptible to transient expression mediated by *Agrobacterium*, that is carried out by injecting a suspension of the desired genes into the leaves using a needle-free syringe. Tobacco plants have been effectively used to reconstruct pathways for various natural compounds, such as lignans, alkaloids, cyanogens, betalains, ketides, and glucosinolates (Miettinen et al., 2014; Law and Sattely, 2015; Rajniak et al., 2015; Polturak et al., 2016; Crocoll et al., 2016; Andersen-Ranberg et al., 2017). Overexpressing terpenoid biosynthesis genes in homologous and ectopic plants, as well as using combinatorial approaches, is thus an important method for maximizing the yield of linalool and costunolide in plants. Overall, here we implemented a green toolkit for the commercialization of several promising plant materials rich in linalool and costunolide. As a result, this provided a potential new route for heterologous plant linalool and costunolide production.

## 2 Materials and methods

### 2.1 Full length candidate genes isolation and cloning from different plant sources

The MEP (1-deoxy-D-xylulose-5-phosphate synthase (DXS), 1-deoxy-D-xylulose 5-phosphate reductoisomerase (DXR), 4-diphosphocytidyl-2-C-methyl-D-erythritol kinase (CMK), isopentenyl-diphosphate isomerase (IDI), geranyl pyrophosphate synthase (GPPS), and linalool synthase (LIS) and MVA (acetoacetyl-CoA-thiolase (AAT), hydroxy-3-methylglutaryl-CoA reductase (HMGR), farnesyl pyrophosphate synthase (FPS), germacrene A synthase (GAS), germacrene A oxidase (GAO), and costunolide synthase (COS) pathway gene sequences were obtained by analyzing a full-length cDNA library from the National Center for Biotechnology Information (NCBI). For each gene, we isolated the cDNA using mRNA from the desired plant species and then cloned it using gene-specific primers (**Supplementary Table 1**). The following conditions were used to perform the PCR reaction: Initial denaturation at 95°C for 5 min, denaturation at 94°C for 30 s, annealing at 56-60°C for 30 or 3 min, extension at 72°C for 2 min for 40 cycles, and final extension at 72°C for 7 min. According to the manufacturer’s instructions, PCR products were cloned into the pENTRTM/TopoR vector (Invitrogen, Carlsbad, CA, USA).

### 2.2 Gene sequence analysis for Moclo golden gate vector construction

To construct the MoClo golden gate system, we first analyzed the cDNA sequence of six MEP and six MVA pathway gene sequences to identify the restriction enzyme sites (BsaI and BpiI) using online Takarabio mutagenesis tools. Among the MEP genes, GPPS does not contain restriction enzyme sites, whereas they are present in the other five genes for either BsaI or BpiI. In the case of MVA genes, AAT does not contain restriction enzyme sites, and the other five genes contain those for either BsaI or BpiI. After analyzing the complete codon sequences of the amino acids, we designed a point mutation primer that specifically targeted the change in a single base pair of both restriction enzymes. Using a new primer, nested PCR was performed, and two separate PCR products were obtained, which were used as templates for PCR to obtain full-length gene products. These products were sequenced to confirm the point mutations.

### 2.3 Transient expression in *N. benthamiana*

The *Agrobacterium tumefaciens* strain GV3101 was heat-shocked to introduce the overexpression constructs for agroinfiltration (Froger and Hall, 2007). Transformed batches were grown in LB medium with carbenicillin (50 mg. L^-1^) and rifampicin (50 mg. L^-1^) for 24 hours at 28°C and 200 rpm. After centrifugation at 4,000 rpm for 10 minutes, cells were resuspended in 10 mM MES buffer containing 10 mM MgCl_2_ and 100 mM acetosyringone (4-hydroxy-3,5-dimethoxyacetophenone, Sigma) to a final OD600 of 0.8, then incubated at room temperature with moderate shaking at 50 rpm for 2 hours. *Agrobacterium* cultures were mixed evenly for co-infiltration such that the gene load was consistent between experiments. Plants for transient transformation of *N. benthamiana* were cultivated in a greenhouse in soil with a minimum of 16 hours of light each day (at 28°C during the day and 25°C at night). By placing a 1 ml syringe without a metal needle against the abaxial side of the leaf and gently injecting the bacterial culture into the leaf, batch samples were infiltrated into the leaves of three-week-old *N. benthamiana* plants. Three individual plants were injected into each construct for the analysis. After agroinfiltration, the plants were grown in a greenhouse for a further five days and then collected for analysis.

### 2.4 RNA isolation from *N. benthamiana* leaves and cDNA synthesis

At five days post (DPI) infiltration, total RNA was extracted from all of the infiltrated leaves. According to the manufacturer’s protocol, RNA was extracted with a Spectrum Plant Total RNA Kit (Sigma-Aldrich, USA). Finally, the pellets were dissolved in distilled water treated with diethyl pyrocarbonate (DEPC). Further, 1 μg of RNA was utilized for cDNA synthesis, which was accomplished using reverse transcription of mRNA with a Bio-Rad cDNA synthesis kit (Bio-Rad Laboratories, Hercules, CA, USA). cDNA synthesis was carried out in a thermal cycler at 72°C for 3 minutes, followed by 4°C cooling of the tube. The master mixture was added to a pre-chilled tube, which was incubated at 42°C for 4 hours. The process was stopped by heating the mixture for 10 minutes at 70°C. The single-stranded cDNA was produced and kept at −20°C until use.

### 2.5 Reverse transcription PCR (RT-PCR) and quantitative Real-Time-PCR (qRT-PCR) analysis

First, we performed reverse transcription PCR (RT-PCR) on individual agro-infiltrated MEP and MVA genes to determine their expression levels. Using specific primers, PCR detection with equivalent cDNA as a template was utilized to determine the levels of expression both MEP and MVA genes (**Supplementary Table 1**). Second, quantitative real-time polymerase chain reaction (qRT-PCR) was executed with a CFX96 Real-Time PCR detection system and the SYBR Premix (Bio-Rad Laboratories, Hercules, CA, USA). The reactions were subjected to the following cycling conditions: denaturation at 95°C for 5 minutes, followed by 45 cycles of denaturation at 95°C for 15 seconds and annealing at 60°C for 30 seconds. Utilizing qRT-PCR primers, both mono- and sesquiterpenoid-targeting genes were amplified. The PrimerQuest Tool (Integrated DNA Technologies, Coralville, IA, USA) was used to design primer pairings. Gene expression was normalized using *N. benthamiana* protein phosphatase 2A (PP2A) (TC21939) as an internal control. The relative quantification approach (ΔΔ^-CT^) was utilized to assess the quantitative variance among replicates. Sequences of primers are listed in **Supplementary Table 1**. For each gene, three biological and three technical replicates were implemented.

### 2.6 Solvent-based extraction of linalool and costunolide from leaf samples

#### 2.6.1 Linalool

Linalool compound extraction was carried out using the method reported by Chang et al. (2020). Briefly, 20 mg of freshly harvested agro-infiltrated leaves were powdered in liquid nitrogen and extracted using 1 ml of 100% hexane. Leaf extract were prepared by briefly vortexing and sonicating for 20 min, then centrifuging at 5,000 rpm for 10 minutes. A fresh tube was used to transfer the supernatant, and it was filtered using a 0.22 µm filter (GHP, 13 mm; Pall, Port Washington, NY, USA). Samples were used for GC-Q-Orbitrap-MS analysis.

#### 2.6.2 Costunolide

Costunolide compound extraction was performed using the method previously reported by Liu et al. (2011). In summary, 100 mg of agro-infiltrated fresh leaves were crushed in liquid nitrogen and extracted using 1 ml dichloromethane. Leaf extract were prepared by quickly vortexed and sonicating for 10 min. Then, they were centrifuged for 15 min at 3,000 rpm, dehydrated using Na_2_SO_4_ for 2 mins and the supernatant was transferred into a new tube, filtered with a 0.22µm filter (GHP, 13 mm; Pall, Port Washington, NY, USA) and used for GC-Q-Orbitrap-MS analysis.

### 2.7 Gas chromatography-Q-orbitrap-mass spectrometry (GC-Q-Oribtrap-MS) analysis

Metabolite qualification and quantification were performed using a GC-Q-Oribtrap-MS system (Q Exactive, Thermo Scientific, USA) equipped with an orbitrap time-of-flight mass spectrometer (Q Exactive, Thermo Scientific, USA) in positive mode, and then peak extraction was conducted with automatic integration software (TraceFinder v1.1; Thermo scientific). The metabolites were measured in the following way: the GC-Q-Orbitrap-MS system was operated by the Thermo scientific software (Xcaliber; Thermo scientific) and a 30 m long, 0.25 mm wide, and 0.25 µm thick reversed-phase column (TG-5MS, Thermoscientific). For the GC-Q-Orbitrap-MS analysis, helium was employed as the carrier gas at a flow rate of 1 ml/min. The injector was set to splitless mode for linalool, with the inlet temperature set to 270°C. The starting oven temperature was 80°C for 2 min, then raised to 160°C after 1 min at a rate of 20°C/min, and held for 8 min at 260°C. The injector was set to splitless mode for costunolide, with the inlet temperature set to 270°C. The starting oven temperature was 80°C for 2 min, then raised to 160°C after 1 min at a rate of 20°C/min and held for 8 min at 260°C. The identification of metabolites was determined on the basis of retention times and mass fragmentation patterns to those of commercial standards, previous studies, a mass spectrometry database such as MASS BANK and NIST08 (National Institute of Standards and Technology, Gaithersburg, MD, USA), and an in-house library. Tracefinder integration software was utilized to automatically extract peak information, including the m/z value, migration time (MT), and peak area. The concentrations of metabolites were measured using linear regression equations obtained from the calibration curves of the respective commercial standards linalool (Sigma-Aldrich: L2602) and costunolide (Sigma-Aldrich: SML0417) (**Supplementary Figure 1**).

## 3 Results

### 3.1 MEP and MVA pathway genes selected for vector construction

**Figure 1:**
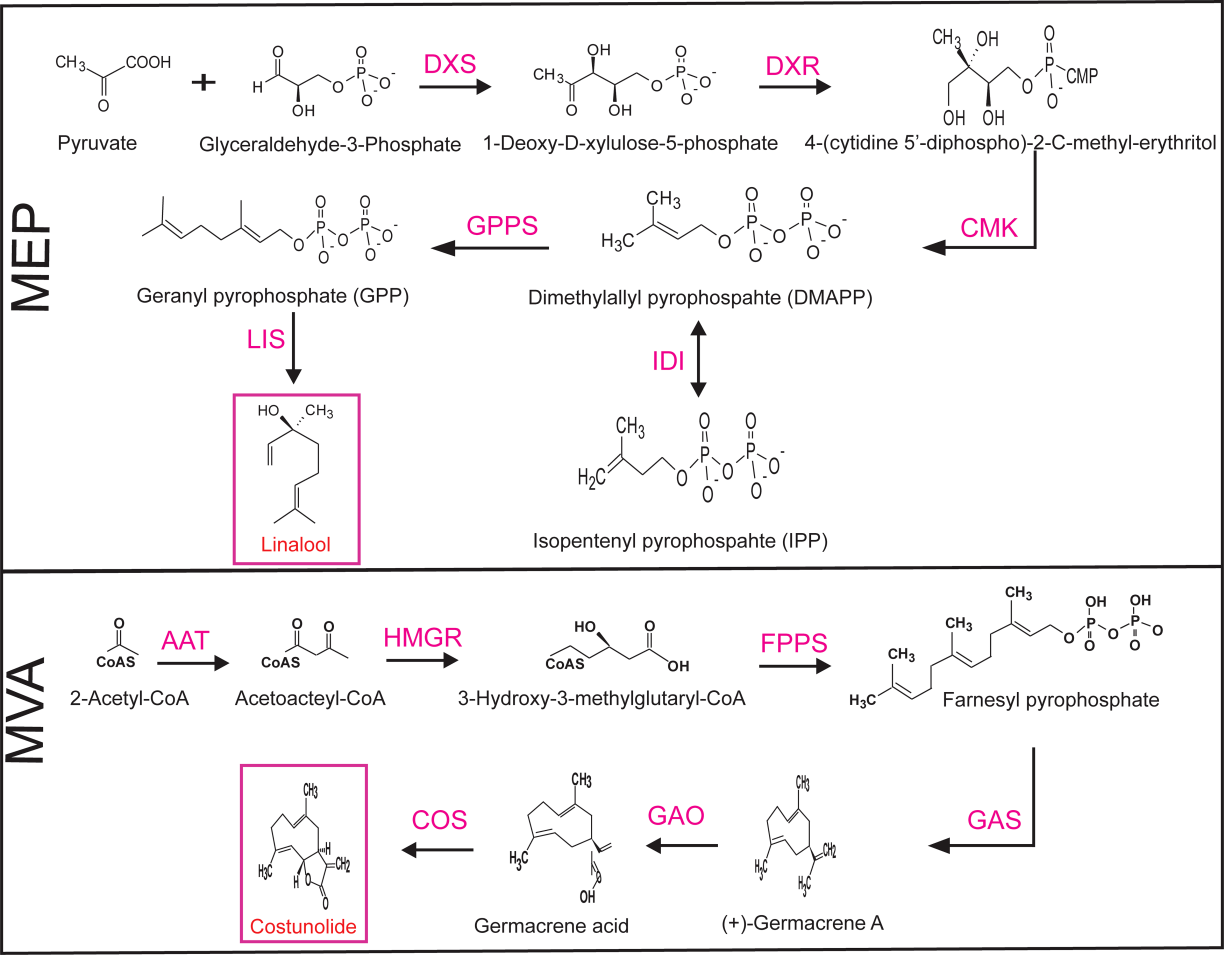
Simplified selected biosynthetic pathway genes of the MEP and MVA pathways. Pink indicates the genes selected in this study. MEP, plastid 2-C-methyl-D-erythritol 4-phosphate; MVA, mevalonate; DXS, 1-deoxy-D-xylulose-5-phosphate synthase; DXR, 1-deoxy-D-xylulose 5-phosphate reductoisomerase; CMK, 4-diphosphocytidyl-2-C-methyl-D-erythritol kinase; IDI, isopentenyl-diphosphate isomerase; GPPS, geranyl pyrophosphate synthase; LIS, linalool synthase; AAT, acetoacetyl-CoA thiolase; HMGR, 3-hydroxy-3-methylglutaryl-CoA reductase; FPS, farnesyl pyrophosphate synthase; GAO, germacrene A oxidase; GAS, germacrene A synthase; COS costunolide synthase.

The biosynthetic pathway of MEP is composed of various enzymes: DXS, DXR, 2-C-methyl-D-erythritol 4-phosphate cytidylyltransferase (CMS), 4-diphosphocytidyl-2-C-methyl-D-erythritol kinase (CMK), 2-C-methyl-D-erythritol 2,4-cyclodiphosphate synthase (MCS), 4-hydroxy-3-methyl-but-2-enyldiphosphate synthase (HDS), 4-hydroxy-3-methyl-but-2-enyldiphosphate reductase (HDR), intermediate isopentenyl-diphosphate isomerase (IDI), and geranyl pyrophosphate synthase (GPPS). The MVA pathway enzymes as follows: acetoacetyl-CoA thiolase (AAT), 3-hydroxy-3-methylglutaryl-CoA synthase (HMGCAS), 3-hydroxy-3-methylglutaryl- CoA reductase (HMGR), mevalonate-5-kinase (MK), phosphomevalonate kinase (PMK), mevalonate-5-phpshate decarboxylase (MPD), mevalonate pyrophosphate decarboxylase (MPPD), and intermediate farnesyl pyrophosphate synthase (FPS) (Mani et al., 2021). In this study, we selected significant biosynthetic, intermediate gene, as well as linalool and costunolide product genes from each pathway as follows: DXS, DXR, CMK, IDI, GPPS, and LIS were selected from the MEP pathway and AAT, HMGR, FPS, GAS, GAO, and COS from the MVA pathway. The simplified MEP and MVA biosynthetic pathways and relevant genes are presented in **Figure 1**. For MEP genes, DXS, DXR, CMK, and IDI gene sequences, and GPPS cDNA sequences were selected from *N. tabacum* and the LIS gene sequence from *Clarkia breweri*. For MVA genes, AAT and FPS gene sequences were selected from *Arabidopsis thaliana*, the HMGR sequence from *N. benthamiana*, GAS and GAO sequences from *Cichorium intybus*, and the COS sequence from *Lactuca sativa*. All candidate gene cDNA sequences were retrieved from GenBank and included: *Nt*DXS, EU650419; *Nt*DXR, DQ839130; *Nt*CMK, KJ159923; *Nt*IDI, AB049815; *Nt*GPPS, KF977582; *Cb*LIS, U58314; *At*AAT, AF364059; *Nb*HMGR, *LC*015758; *At*FPS, NM117823; *Ci*GAS, AF497999; *Ci*GAO, GU256644; and *Ls*COS, HQ439599.

### 3.2 MoClo golden gate level-0 and level-1 vector map of MEP and MVA pathway genes and PCR products

**Figure 2:**
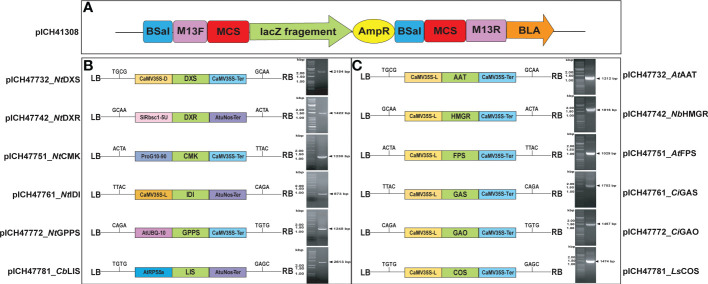
MoClo golden gate vector construction map. **(A)** Level-0 (Entry level vector), **(B, C)**. MVA and MEP pathway gene constructs and PCR amplification of gel products (AAT, 1.2 kb; HMGR, 1.8 kb; FPS, 1.0 kb; GAS, 1.7 kb; GAO, 1.4 kb and COS, 1.4 kb, DXS, 2.1 kb; DXR, 1.4 kb; CMK, 1.2 kb; IDI, 0.8 kb; GPPS, 1.2 kb; and LIS, 2.6 kb) were isolated and cloned into the MoClo golden gate vector. The expression cassette is located between the left and right T-DNA boarders (LB and RB, respectively) and contain**s** the cauliflower mosaic virus 35S promoter and *Arabidopsis* ubiquitin promoter (UBQ), respectively. Along with genes harboring the CaMV 3′ UTR and nopaline synthase terminators, the desired cDNAs of MEP and MVA pathway genes were under the control of the *Arabidopsis* ubiquitin promoter with the 35S terminator.

To construct the Moclo golden gate level-1 vector, first, the full-length cDNAs of MEP (DXS, DXR, CMK, IDI, GPPS, and LIS) and MVA (AAT, HMGR, FPS, GAS, GAO, and COS) pathway genes were subcloned into a MoClo golden gate entry level-0 (pICH41308) vector. The level-1 module of MEP genes named as (pICH47732_ *Nt*DXS, pICH47742_*Nt*DXR, pICH47751_*Nt*CMK, pICH47761_ *Nt*IDI, pICH47772_ *Nt*GPPS, and pICH47781_*Cb*LIS) MVA genes (pICH47732_*At*AAT, pICH47742_*Nb*HMGR, pICH47751_*At*FPS, pICH47761_*Ci*GAS, pICH47772_*Ci*GAO, and pICH47781_*Ls*COS) was assembled using the level-0 module including the CDS of each gene, the Bsa I restriction enzyme, and T4 ligase through the promoter and terminator. In the Lv.1 module, different types of promoters (*CaMV35S*-L, *CaMV35S*-D, *Sl*Rbsc1-5U, ProG10-90, *At*UBQ-10, and *At*RPS5a) and terminators (CaMV35S-Ter and AtuNos-Ter) were used, as a previous report showed that suppression of expression may occur when the same promoter is used consecutively in the level-1 module (Tian et al., 2022). The list of MoClo vector promoters and terminators is presented in **Supplementary Table 2**. Each level-1 backbone vector consists of a 4-overhang sequence, so that the promoter-CDS-terminator can be cleaved and linked in one step when the type IIS restriction enzyme is used (**Figure 2**). The primers used to construct the level-0 vector are listed in **Supplementary Table 1**. Each of the generated vectors was then transformed into *A. tumefaciens* GV3101, and each cloned gene was agro-infiltrated individually and/or in combination with other genes into *N. benthamiana* (**Figure 2**).

### 3.3 MEP and MVA pathway gene expression analysis using RT-PCR and qRT-PCR

**Figure 3:**
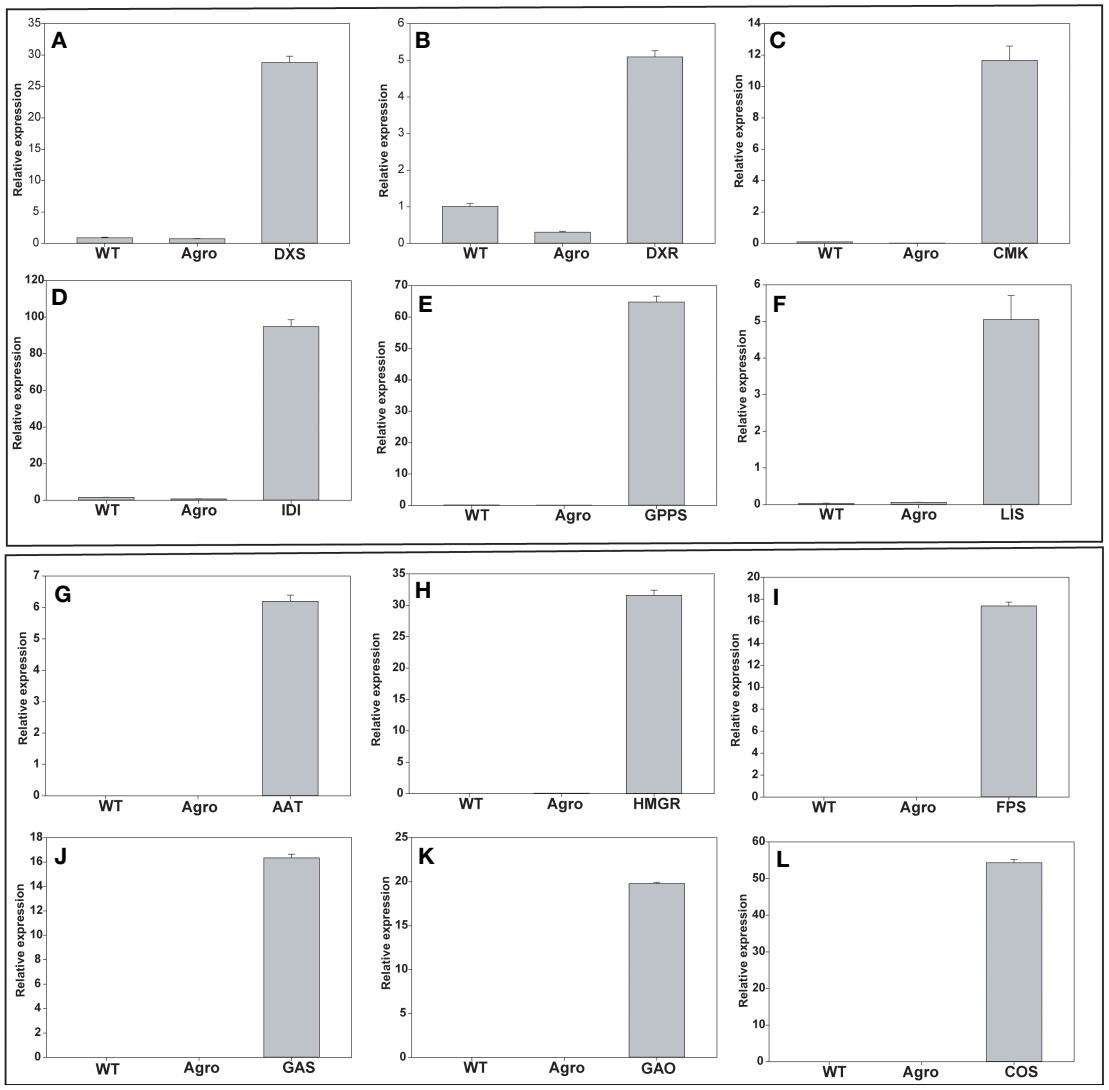
mRNA overexpression analysis of MEP and MVA pathway genes using qRT-PCR. **(A)** DXS, **(B)** DXR, **(C)** CMK, **(D)** IDI, **(E)** GPPS **(F)** LIS, **(G)** AAT, **(H)** HMGR, **(I)** FPS, **(J)** GAS, **(K)** GAO, and **(L)** COS. Gene expression levels were normalized to those of PP2A and their relative expression is shown. MEP, plastid 2-C-methyl-D-erythritol 4-phosphate; MVA, mevalonate; DXS, 1-deoxy-D-xylulose-5-phosphate synthase; DXR, 1-deoxy-D-xylulose 5-phosphate reductoisomerase; CMK, 4-diphosphocytidyl-2-C-methyl-D-erythritol kinase; IDI, isopentenyl-diphosphate isomerase; GPPS, geranyl pyrophosphate synthase; LIS, linalool synthase; AAT, acetoacetyl-CoA thiolase; HMGR, 3-hydroxy-3-methylglutaryl-CoA reductase; FPS, farnesyl pyrophosphate synthase; GAO, germacrene A oxidase; GAS, germacrene A synthase; COS costunolide synthase; PP2A, protein phosphatase 2A.

The expression levels of genes involved in the MEP (DXS, DXR, CMK, IDI, GPPS, and LIS) and the MVA pathway (AAT, HMGR, FPS, GAS, GAO, and COS) were analyzed using RT-PCR and qRT-PCR. Analyses were performed to quantify the endogenous gene expression in leaf samples at five DPI. First, RT-PCR and cDNA were used to amplify the full-length genes to confirm the real-time level of gene expression. Wildtype (WT) and *Agrobacterium tumefaciens* GV3101 (Agro) were negative controls. DNA gel electrophoresis was used to examine transgene expression levels (**Supplementary Figure 2**). Second, qRT-PCR analysis was performed, and the relative expression levels of each gene were found to be relatively higher than those in the control plants (**Figure 3**). The amplification efficiencies of each individual gene and that of the reference gene protein phosphatase 2A (PP2A) were found to be higher compared to those in non-infiltrated leaf samples. *N. benthamiana* PP2A (TC21939) primers were used as internal controls for gene expression normalization. Our results revealed that the MEP pathway genes DXS, DXR, CMK, IDI, GPPS, and LIS were 28.8-, 5.09-, 11.65-, 94.87-, 64.76-, and 5.05- fold upregulated, respectively. In the case of MVA pathway genes, AAT, HMGR, FPS, GAS, GAO, and COS were 6.1-, 31.5-, 17.3-, 16.3-, 19.7- and 54.3-fold upregulated, respectively. Quantitative analysis of the 12 genes revealed that the transcript level at 5 DPI was approximately 5- to 94-fold higher than that in control leaf samples (**Figure 3**). The overall qPCR real time raw data file for MEP and MVA genes were provided in supplementary excel file.

### 3.4 Linalool and costunolide production in *N. benthamiana* leaves

**Figure 4:**
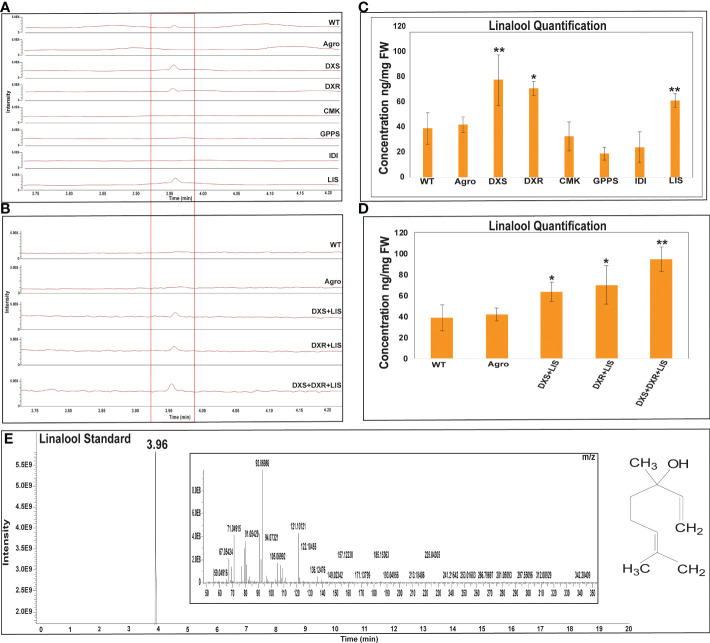
GC-Q-Oribtrap-MS linalool profiling and quantification for individual and combinatorial approaches. **(A, B)** Individual and combinatorial linalool metabolite profiling. **(C, D)** Individual and combinatorial costunolide quantification. **(E)** Linalool standard metabolite profiling and m/z ions. Quantification of each bar represents the mean ± standard deviation (SD) of three independent experiments with three technical replicates. Statistical analyses (t-test) were conducted using SigmaPlot 12.5. For t-test analysis, the wild type values were used for construct comparisons. * indicates significant differences compared with the wild-type (*p < 0.05, **p < 0.01).

**Figure 5:**
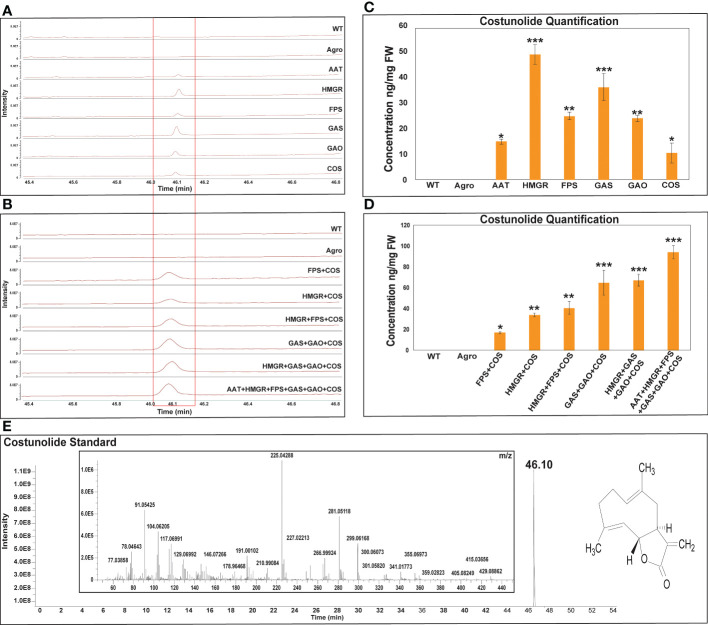
GC-Q-Oribtrap-MS costunolide profiling and quantification for individual and combinatorial approaches. **(A, B)** Individual and combinatorial costunolide metabolite profiling. **(C, D)** Individual and combinatorial costunoilde quantification. **(E)** Costunolide standard metabolite profiling and m/z ions. Quantification of each bar represents the mean ± standard deviation (SD) of three independent experiments with three technical replicates. Statistical analyses (t-test) were conducted using SigmaPlot 12.5. For t-test analysis, the wild type values were used for construct comparisons. * indicates significant differences compared with the wild-type (*p < 0.05, **p < 0.01, ***p < 0.01).

The linalool and costunolide peak was identified by comparing its GC-Q-Orbitrap-MS profile with that of the authentic linalool and costunolide standard. For linalool and costunolide quantification, multiple-point injection concentrations were used (**Supplementary Figure 1**). The infiltrated leaf samples were subjected to GC-Q-Oribtrap-MS analysis for linalool and costunolide profiling (**Figure 4**). In terms of absolute quantification, GC-Q-Orbitrap-MS results revealed that linalool production in the DXS, DXR, and LIS constructs ranged between 70-77 ng.mg^-1^, while that in the CMK, GPPS, and IDI constructs ranged between 19-32 ng.mg^-1^. The individual DXS constructs produced a maximum of 77 ng.mg^-1^ of linalool. Second, the expression levels of the rate-limiting DXS and DXR genes in the MEP pathway were examined. Linalool production was improved using a combinatorial construct. The DXS+LIS construct produced 63 ng.mg^-1^ linalool whereas DXR+LIS produced 69 ng.mg^-1^. The DXS+DXR+LIS construct was accompanied by an increase in linalool production of 94 ng.mg^-1^ in the fresh weight of *N. benthamiana* leaf samples, whereas in the control sample, WT and Agro produced 38 and 41 ng.mg^-1^, respectively (**Figure 4**). Similarly, the costunolide production in the AAT and COS constructs was in the range of 10-14 ng.mg^-1^. That of the GAO, FPS, GAS, and HMGR constructs was between 23-48 ng.mg^-1^ for individual constructs. The individual HMGR constructs produced a maximum of 48 ng.mg^-1^ of costunolide. In addition, we analyzed the expression levels of the rate-limiting gene encoding HMGR in the MVA pathway and those of the sesquiterpenoid cyclization genes GAS and GAO. Costunolide production in combinatorial constructs improved in a stepwise manner. The FPS+COS, HMGR+COS, and HMGR+FPS+COS constructs produced costunolide in the range of 16-40 ng.mg^-1^. The costunolide production of the GAS+GAO+COS construct was 64 ng.mg^-1^ and that of the HMGR+GAS+GAO+COS construct was 67 ng mg^-1^. Interestingly, the combination of six genes (AAT+HMGR+FPS+GAS+GAO+COS) was accompanied by an increase of 94 ng.mg^-1^ costunolide in the fresh weight of *N. benthamiana* leaf samples, whereas costunolide was not produced in the control sample WT and Agro (**Figure 5**). Overall, linalool and costunolide metabolite concentration of individual and combinatorial were listed in **Supplementary Table 3**.

## 4 Discussion

Synthetic metabolic engineering of plants is an emerging field for producing beneficial compounds. It relies on the identification of key synthase genes and regulatory mechanisms for the biosynthetic pathway of target compounds, and it is crucial to establish the ability of the organism to efficiently stack and transform multiple genes. Establishing a multigene stacking strategy typically necessitates the incorporation of one or more exogenous genes into the heterologous host-genome. The assembly of multiple DNA fragments has made significant strides in recent decades (Gutensohn and Dudareva, 2016; Nazhand et al., 2020; Firsov et al., 2020; Breitel et al., 2021; Grützner et al., 2021). Linalool and costunolide are the precursors of many biologically active mono- and sesquiterpenoids, respectively. For commercial manufacture of these compounds, reconstitution of the biosynthetic pathway in heterologous hosts might be an attractive approach. In this study, MEP (DXS, DXR, CMK, IDI, GPPS and LIS) and MVA (AAT, HMGR, FPS, GAS, GAO and COS) (**Figure 1**) pathway genes were cloned and agro-infiltrated in *N. benthamiana* and the two major classes of terpenoid compounds linalool and costunolide were quantified.

Golden gate cloning is used to assemble several DNA fragments in a recipient vector in a specific linear order using a one-pot assembly technique. The golden gate cloning principle uses a type-IIS restriction enzyme and ligase in a single step of restriction-ligation to assemble numerous DNA fragments in a vector in a predetermined linear order (Marillonnet and Grützner, 2020). In our study, we utilized MoClo golden gateway system and constructed selected genes in linear order with each full-length cDNAs and subcloned using primers at level 0 (**Figure 2**). All genes were agro-infiltrated into *N. benthamiana.* Quantification of the expression of the cloned MEP and MVA biosynthesis genes by qRT-PCR (**Figure 3**) to determine the effectiveness of the individual and combinatorial agroinfiltration systems.

The attempting to rebuild of pathways in the transient *Nicotiana* spp. plant expression system for many therapeutically important proteins has been established, and this system has been found to be an excellent model for studying the production of mono- and sesquiterpenoid pharmaceutical compounds. However, the majority of plant flux engineering strategies have been on either overexpressing rate-limiting enzymes in the MEP or MVA pathway, such as DXS and HMGR, or transferring metabolic enzymes to IPP- and FPP-rich organelles, such as plastids and mitochondria. (Reed and Osbourn, 2018). The present work, however, aimed to compare individual and combinatorial system strategies of selected enzyme activities to increase linalool and costunolide production. One important insight by this work is that when rate-limiting enzymes (DXS and HMGR) were combined with key enzyme-targeted metabolites, linalool and costunolide production was high.

A previous study of monoterpenoid production by overexpression of MEP genes and transgenic plant constructs revealed that the 1^st^ committed MEP pathway enzyme, DXS, increased the accumulation of end product production of monoterpenes (Zhao et al., 2020). Geranyl diphosphate synthase small subunit I (GPS SSU1) overexpression in Lour (*Listea cubeba*) plants indicated a substantial increase in the levels of monoterpenes. Two significant MEP pathway genes, DXS and DXR, were cloned and expressed using transgenic tobacco plants, leading to an increase in the quantity of the monoterpenoid linalool (Zhang et al., 2018). Likewise, the co-expression of Mentha *X* Piperita GPS SSU, *Arabidopsis thaliana* GPS1, and *Solanum lycoperscum* DXP *via* transient expression in *N. benthamiana* plants improved the synthesis of monoterpenes, including myrcene, alpha/beta-pinene, linalool, and limonene (Kang et al., 2015). *S*-linalool synthase overexpression in petunia led to the monoterpene synthesis of freshly generated linalool. Monoterpene concentrations were elevated as a consequence of overexpression of the *S*-linalool synthase gene in tomatoes (*Lycopersicon esculentum*) (Lewinsohn et al., 2001). In our study, the agro-infiltrated leaves with the monoterpenoid MEP rate-limiting enzymes DXS and DXR produced linalool in the range of 70-77 ng.mg^-1^ whereas in combination with linalool synthase (DXS+DXR+LIS) they produced a maximum of 94 ng.mg^-1^ in *N. benthamiana* (**Figure 4**).

Several medicinal plants have been found to contain costunolide, which is linked to many biological activities, which includes anti-fungal, anti-viral, anti-carcinogenic, and immunosuppressive activities. Many studies have reported sesquiterpenoid production in plants. *Artemisia annua* beta-caryophyllene synthase was transferred into *Agrobacterium* through a viral vector in a previous study, and then agroinfiltrated into *N. benthamiana* leaves to produce 26.5 mg of β-caryophyllene (Muthusamy et al., 2020). Transient co-expression of the parthenolide pathway candidate genes in *N. benthamiana* was used to reconstitute the co-expression, and up to 1.4 µg of the final product was generated (Liu et al., 2014). Different plant-specific gene co-expressions, including chicory costunolide synthase (COS), chicory GAO, and feverfew GAS, were agro-infiltrated and reconstituted in *N. benthamiana*, with findings demonstrating the synthesis of 60 ng.g^-1^ of costunolide (Liu et al., 2011). In our study, agro-infiltrated leaves with the sesquiterpenoid MVA rate-limiting enzyme HMGR produced costunolide at 48 ng.mg^-1^. When the rate-limiting enzyme was combined with the key enzyme (HMGR+GAS+GAO+COS) construct, 67 ng mg^-1^costunolide was produced. Finally, the combination of six genes (AAT+HMGR+FPS+GAS+GAO+COS) was accompanied by an increase of 94 ng.mg^-1^ costunolide in the fresh weight of *N. benthamiana* leaf samples (**Figure 5**).

The main goal of this study was to implement a MoClo golden gate cassette system for expressing selected MEP and MVA pathway genes from different plant host sources to be further used for system biology applications or studies. In *A. annua*, several sesquiterpene synthases compete for FPP. These include amorpha-4,11-dienesynthase (ADS), epi-cedrol synthase, β-caryophyllene synthase, (E)-farnesene synthase, germacrene A synthase, and squalene synthase. ADS is the only enzyme involved in artemisinin production (Liu et al., 2011; Kanagarajan et al., 2012; Cankar et al., 2015; Muthusamy et al., 2020). Numerous studies have been conducted to increase mono- and sesquiterpene biosynthesis by inhibiting the expression of mono- and sesquiterpene synthase(s) striving for GPPS and FPP using antisense technology or RNA interference (Li et al., 2020). The findings of this study will help us to effectively block mono- and sesquiterpene synthase(s), which may redirect metabolic flux and increase linalool and costunolide production in the future. Additionally, microbial production technologies, such as modified yeast and bacteria, have also shown promise for meeting the linalool and costunolide demand. The formation of inclusion bodies, however, is a significant drawback in the production of microbial expression systems (Zhou et al., 2020). This highlights the importance of investigating other host systems for native protein synthesis. Modern research on *N. benthamiana* transient heterologous expression have highlighted the rising popularity of plant-based expression technologies for terpenoid production.

Overall, our findings show that expressing critical MEP and MVA biosynthesis pathway genes in single or combined systems will further enable industrial-scale production of linalool and costunolide in heterologous plant systems. Its success will boost the availability of linalool and costunolide with enhanced chemical features, speeding up their exploration.

## Data availability statement

The original contributions presented in the study are included in the article/**Supplementary Materials**. Further inquiries can be directed to the corresponding authors.

## Author contributions

VM, SP, and KL conceived and designed the conceptualization; SP and VM performed experiments; JK and SL provided feedback and helped to improve final manuscript; VM and SP wrote the manuscript. All authors have read and agreed to the published version of the manuscript.
