# Supplementary material for: Combinatorial transient gene expression strategies to enhance terpenoid production in plants
Source: Front Plant Sci. 2022 Dec 13;13:1034893. doi: 10.3389/fpls.2022.1034893 (PMC9793405; doi:10.3389/fpls.2022.1034893)
Supplement: Supplementary file 1 [file DataSheet_1.pdf]

Supplementary Table 1. List of MEP and MVA gene name, accession number, plant source, full-length, qPCR primer sequences and gene products.

Pathways	Gene name	Accession number	Plant source	Primer Name	Full length sequence (5´ to 3´)	Size (bp)	qPCR sequence (5´ to 3´)	Size (bp)
MEP	1-deoxy-D-xylulose-5-phosphate synthase (DXS)	EU650419	*Nicotiana tabacum*	DXS_FP	ATGGCGCTGAATTTGCTG	1212	GCAGACAAGTATCACGGAGTAG	99
				DXS_RP	TTATACAAGAGCTGGACTAAAACC		CTCCGCAAAGTACGTCGTATAG	
	1-deoxy-D-xylulose 5-phosphate reductoisomerase (DXR)	DQ839130	*Nicotiana tabacum*	DXR_FP	ATGGCGCTGAATTTGCTG	1816	CCTACAGTGGCTGCCATAAA	94
				DXR_RP	TTATACAAGAGCTGGACTAAAACC		GCGCAAGAGGAAGAACAAATG	
	4-diphosphocytidyl-2-C-methyl-D-erythritol kinase (CMK)	KJ159923	*Nicotiana tabacum*	CMK_FP	ATGGCTTCTTGTAATTTTCTAAG	1029	TCTGGTGAGATCGGTTCTGATA	100
				CMK_RP	CTAAGAATTATCAAAAGATCCGG		GTATGGGCGATGGGATATCTTG	
	Isopentenyl-diphosphate isomerase	AB049815	*Nicotiana tabacum*	IDI_FP	ATGTCGCTGACAACTACG	1753	GCAACGATCAGCAACAAAGG	106
				IDI_RP	TTAAGTCAATTTGTGGA		CCCAAGAGCATTCTCC	

	(IDI)				TGGTTT		TCAAT	
	Geranyl pyrophosphate synthase (GGPS)	KF977582	*Nicotiana tabacum*	GPPS_FP	ATGCTATTTGCAAGGGG	1468	GTACCAGGCAGCAGTGTATAG	93
				GPPS_RP	CTATTTTGTTCTTGTGATGACTC		GTCGTGTGTCAGCATCATCTA	
	Linalool synthase (LIS)	U58314	*Clarkia breweri*	LIS_FP	ATGCAGCTCATAACAAATTTCTC	1474	CTGCGGAACTATACAGGGATTC	108
				LIS_RP	TTAACTGAAACATAGTTTGATGTTG		CACGGATTTCGTCGTCATCTAA	
MVA	Acetoacetyl-CoA-thiolase (AAT)	AF364059	*Arabidopsis thaliana*	AAT_FP	ATGGCCCATACATCAGAATCTG	1212	TTTGGCAGAAGCAAGGAAGG	103
				AAT_RP	TCAAAGGAGCTCAAGAACTAGAG		TCCCATCCCACAGTCGTTATAG	
	Hydroxy-3-methylglutaryl-CoA reductase (HMGR)	LC015758	*Nicotiana benthamiana*	HMGR_FP	ATGGACGTTCGCCGGAGATC	1816	CGTGCAGATACCTGTTGGAATA	100
				HMGR_RP	TTAGGAGGATGCCTTTGTGACATC		TGGTGCTAGCCACTAAACATC	
	Farnesyl pyrophosphate synthase (FPS)	NM117823	*Arabidopsis thaliana*	FPS_FP	ATGAGTGTGAGTTGTTGTTGTAG	1029	TACGCGGAGGGAAGCTAAA	109
				FPS_RP	CTACTTCTGCCTCTTGTAGATCT		CCAAGAGCACATGAGAGGAAAG	

	Germacrene A synthase (GAS)	AF497999	*Cichorium intybus*	GAS_FP	ATGGCTCTCGTTAGAAACAA	1753	ATTCCAACAATCCGTCCCTTAC	101
				GAS_RP	TCAGTTTTCGAGACTCGGTGGA		TGATACGTGCCCGAGAGTAATA	
	Germacrene A oxidase (GAO)	GU256644	*Cichorium intybus*	GAO_FP	ATGGAGCTCTCACTCACTAC	1468	AGGGCTAGGCTAACCAGTAT	120
				GAO_RP	TTAAAAACTTGGTACGAGTATCAA		CCGTAGCAACACATCGAGAA	
	Costunolide synthase (COS)	HQ439599	*Lactuca sativa*	COS_FP	ATGGAGCCTCTCACCATCG	1474	CAAAGCCGTTGTTCTGGATATG	123
				COS_RP	CTAGGACTTGAGGATCGGGA		CTCACTTCTTCTTGTGCTCTCT	

Supplementary figure 1: Linalool and costunolide linearity graph with three point-injection.

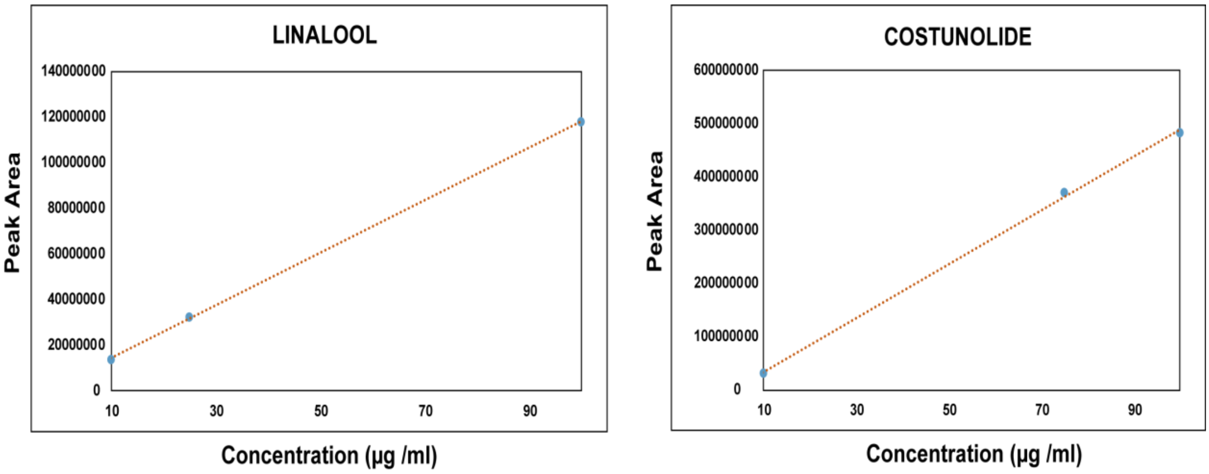

Supplementary figure 2: Full-length RT-PCR analysis for MEP and MVA pathway gene expression analysis.

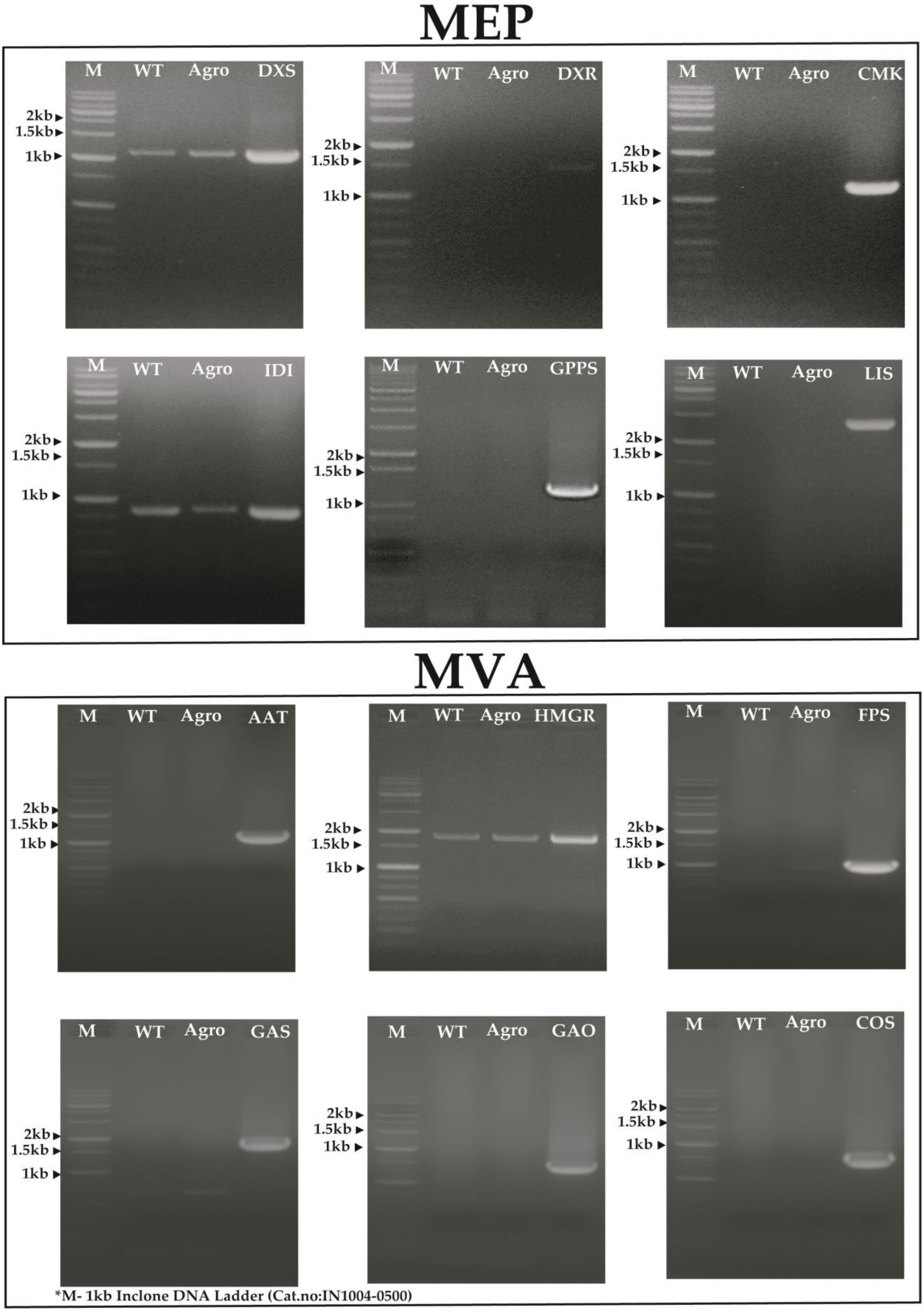

Supplementary Table 2. List of MoClo golden gate vector promoter and terminator

Promoter/Terminator	Full name
CaMV35S-D	Promoter (double), 35s (Cauliflower Mosaic Virus) + 5'UTR, Ω (Tobacco Mosaic Virus)
SIRbsc1-5U	Promoter and 5'UTR, RbcS2 (*S. lycopersicum*)
ProG10-90	Promoter G10-90 (strong, constitutive in *Arabidopsis*)
CaMV35S-L	promoter (0.4 kb), 35s (Cauliflower Mosaic Virus) + 5'UTR, Ω (Tobacco Mosaic Virus)
AtUBQ-10	GGAG_Pro-AtuNos_5U-TMV_AATG promoter, nos, (*Agrobacterium tumefaciens*) + 5'UTR, Ω (Tobacco Mosaic Virus)
AtRPS5a	~ 1700 bp upstream of *Arabidopsis* RPS5a (At3g11940) (strong, constitutive promoter in *Arabidopsis*)
CaMV35S-Ter	3'UTR, polyadenylation signal/terminator, 35s (Cauliflower Mosaic Virus)
AtuNos-Ter	3'UTR, polyadenylation signal/terminator, nos (*A. tumefaciens*)

Supplementary Table 3: Linalool and costunolide metabolite concentration in individual and combinatorial constructs

Pathway	Constructs	Metabolite concentration (ng/mg)
MEP (Linalool-Individual and Combinatorial)	WT	38.84
	Agro	41.00
	DXS	77.43
	DXR	70.73
	CMK	32.61
	GPPS	19.04
	IDI	23.99
	LIS	70.85
	DXS+LIS	63.47
	DXR+LIS	69.99
	DXS+DXR+LIS	94.55
MVA (Costunolide-Individual and Combinatorial)	WT	0.00
	Agro	0.00
	AAT	14.87
	HMGR	48.71
	FPS	24.73
	GAS	36.02
	GAO	23.86
	COS	10.48
	FPS+COS	16.93
	HMGR+COS	33.89
	HMGR+FPS+COS	40.63
	GAS+GAO+COS	64.84
	HMGR+GAO+GAS+GAO+COS	67.22
	AAT+HMGR+FPS+GAS+GAO+COS	94.18
